# Mild increases in plasma creatinine after intermediate to high-risk abdominal surgery are associated with long-term renal injury

**DOI:** 10.1186/s12871-021-01353-2

**Published:** 2021-04-30

**Authors:** Alexandre Joosten, Brigitte Ickx, Zakaria Mokhtari, Luc Van Obbergh, Valerio Lucidi, Vincent Collange, Salima Naili, Philippe Ichai, Didier Samuel, Antonio Sa Cunha, Brenton Alexander, Matthieu Legrand, Fabio Silvio Taccone, Anatole Harrois, Jacques Duranteau, Jean-Louis Vincent, Joseph Rinehart, Philippe Van der Linden

**Affiliations:** 1 Department of Anesthesiology, CUB Erasme, Université Libre de Bruxelles, 808 route de Lennik, 1070 Bruxelles, Belgium; 2 Department of Anesthesiology and Intensive Care, Hôpitaux Universitaires Paris-Sud, Université Paris-Sud, Paul Brousse Hospital, Assistance Publique Hôpitaux de Paris (APHP), Université Paris-Saclay, 12 Avenue Paul Vaillant Couturier, 94800 Villejuif, France; 3 Department of Hepato-biliary Surgery, CUB Erasme, Université Libre de Bruxelles, 808 route de Lennik, 1070 Bruxelles, Belgium; 4 Department of Anesthesiology, Médipole, Lyon Villeurbanne France; 5 Department of Liver Intensive Care Unit, AP-HP, Assistance Publique Hôpitaux de Paris, Paul-Brousse Hospital, Centre Hépato-Biliaire, 12 Avenue Paul Vaillant Couturier, 94800 Villejuif, France; 6 Department of Hepato-biliary and Pancreatic Surgery, Assistance Publique Hôpitaux de Paris, Paul-Brousse Hospital, Centre Hépato-Biliaire, 12 Avenue Paul Vaillant Couturier, 94800 Villejuif, France; 7 Department of Anesthesiology, University of California San Diego, 9500 Gilman Dr, La Jolla, CA 92093 USA; 8 Department of Anesthesia and Perioperative care, University of California, San Francisco, 500 Parnassus Avenue, San Francisco, USA; 9 UMR INSERM 942, Institut National de la Santé et de la Recherche Médicale (INSERM), INI-CRCT network, Paris, France; 10 Department of Intensive Care, CUB Erasme, Université Libre de Bruxelles, 808 route de Lennik, 1070 Bruxelles, Belgium; 11 Department of Anesthesiology and Intensive Care, Hôpitaux Universitaires Paris-Sud, Université Paris-Sud, Bicetre Hospital, Assistance Publique Hôpitaux de Paris (APHP), Université Paris-Saclay, 78 rue du General Leclerc, 94270 Le Kremlin Bicetre, France; 12 Department of Anesthesiology and Perioperative Care, University of California Irvine, 101, the City Drive South, Orange, California, USA; 13 Department of Anesthesiology, Brugmann Hospital, Université Libre de Bruxelles, 4, Place A. Van Gehuchten, 1020 Bruxelles, Bruxelles, Belgium

**Keywords:** Acute kidney disease, Chronic kidney disease, Perioperative, Dialysis, Follow-up, Postoperative complications

## Abstract

**Background:**

The potential relationship between a mild acute kidney injury (AKI) observed in the immediate postoperative period after major surgery and its effect on long term renal function remains poorly defined. According to the “Kidney Disease: Improving Global Outcomes” (KDIGO) classification, a mild injury corresponds to a KIDIGO stage 1, characterized by an increase in creatinine of at least 0.3 mg/dl within a 48-h window or 1.5 to 1.9 times the baseline level within the first week post-surgery. We tested the hypothesis that patients who underwent intermediate-to high-risk abdominal surgery and developed mild AKI in the following days would be at an increased risk of long-term renal injury compared to patients with no postoperative AKI.

**Methods:**

All consecutive adult patients with a plasma creatinine value ≤1.5 mg/dl who underwent intermediate-to high-risk abdominal surgery between 2014 and 2019 and who had at least three recorded creatinine measurements (before surgery, during the first seven postoperative days, and at long-term follow up [6 months-2 years]) were included. AKI was defined using a “modified” (without urine output criteria) KDIGO classification as mild (stage 1 characterised by an increase in creatinine of > 0.3 mg/dl within 48-h or 1.5–1.9 times baseline) or moderate-to-severe (stage 2–3 characterised by increase in creatinine 2 to 3 times baseline or to ≥4.0 mg/dl). The exposure (postoperative kidney injury) and outcome (long-term renal injury) were defined and staged according to the same KDIGO initiative criteria. Development of long-term renal injury was compared in patients with and without postoperative AKI.

**Results:**

Among the 815 patients included, 109 (13%) had postoperative AKI (81 mild and 28 moderate-to-severe). The median long-term follow-up was 360, 354 and 353 days for the three groups respectively (*P* = 0.2). Patients who developed mild AKI had a higher risk of long-term renal injury than those who did not (odds ratio 3.1 [95%CI 1.7–5.5]; *p* < 0.001). In multivariable analysis, mild postoperative AKI was independently associated with an increased risk of developing long-term renal injury (adjusted odds ratio 4.5 [95%CI 1.8–11.4]; *p* = 0.002).

**Conclusions:**

Mild AKI after intermediate-to high-risk abdominal surgery is associated with a higher risk of long-term renal injury 1 y after surgery.

## Background

Acute kidney injury (AKI) occurs frequently in patients after major surgeries [1–3]. Most data regarding AKI outcomes have come from critically ill patients or postoperative vascular and cardiac patients [4–12]. In these study populations, AKI has consistently been reported to be associated with increased lengths of hospital stay, higher readmission rates and greater healthcare costs [12–15]. Development of AKI in these patients has also been associated with altered short and long-term clinical outcomes, including death [10, 16].

One of the most common systems used to diagnose AKI is the “Kidney Disease: Improving Global Outcomes” (KDIGO) classification, in which kidney dysfunction is based on changes in serum creatinine and urine output [17]. However, as urine output is rarely accurately measured in the perioperative setting, postoperative AKI is frequently assessed based on an increase in serum creatinine alone. Mild kidney injury is more frequent than moderate or severe injury after a major surgical procedure. Unfortunately, these three stages are generally combined into a global composite of “AKI”, ignoring the obvious differences in incidence and severity across the stages. Moreover, many clinicians (surgeons, intensivists and anaesthesiologists), routinely under-recognise the importance of mild AKI, [18] as they often consider postoperative AKI to be a transient phenomenon without short or long-term consequences [19]. However, Turan et al. recently reported that mild postoperative AKI could affect long-term renal function in patients who had had various non-cardiac surgical procedures [20]. Nevertheless, there is limited specific literature regarding the long-term renal consequences of mild AKI after major abdominal surgery [19–21].

We therefore conducted a retrospective cohort study to analyse the association between postoperative AKI and long-term renal injury after intermediate-to high-risk abdominal surgery. We hypothesised that patients with a slight increase in their postoperative plasma creatinine, corresponding to mild AKI, would be at higher risk of long-term renal injury compared to patients without postoperative creatinine increase.

## Methods

The Ethics Committee of Erasme, Free university of Brussels, Belgium on February 10th, 2020, approved this single centre retrospective cohort analysis (committee’s reference number: P2020/031). Data collection was performed by Z. M in our institution between February 11th and April 1st 2020.

We included all consecutive adult patients (≥ 18 years old) who:

1. had undergone elective intermediate-to high-risk open abdominal surgery (including hepatobiliary surgery, pancreatectomy, gastrectomy, oesophagectomy, cancer debulking, cystectomy) under general anaesthesia between January 1st, 2014, and April 30th, 2019. Patients who had had major vascular surgery were also included if the surgery involved an abdominal incision (e.g., aorto bifemoral bypass and abdominal aortic aneurysm surgery);
2. had a plasma creatinine value measured before surgery, within 7 days after surgery, and at a later follow up visit (6 months to 2 years after surgery).

Patients who received dialysis in the preoperative period, those with chronic kidney disease (predefined as a baseline creatinine level > 1.5 mg/dl), those who had emergency surgery and patients who had another surgical procedure in the 2 y following their first surgery (unless it was a redo surgery in the same admission) were excluded. Patients who had suprarenal clamping during their vascular surgery were also excluded as this clamping phase can seriously impact renal function.

For each eligible patient, we recorded, from our hospital health records, the plasma creatinine concentration prior to surgery (the most recent result available in the 3 months before surgery), the highest creatinine concentration during the seven postoperative days, and the creatinine concentration at long-term follow up (between 6 months and 2 years; if multiple creatinine values were available, the measurement closest to 1 y following surgery was always selected). If no long-term creatinine measurement was available in the hospital database, attempts were made to contact the patients and/or their general practitioners to obtain any values that had been measured elsewhere.

The change in creatinine concentration between the preoperative and the postoperative period was used to classify patients according to a “modified” KDIGO classification in which the urine output criteria were not considered [17]. Mild AKI (KDIGO stage 1) was characterised by an increase in creatinine of > 0.3 mg/dl within 48-h or 1.5–1.9 times baseline; moderate AKI (KDIGO stage 2) by an increase in creatinine of 2–2.9 times baseline; and severe (KDIGO stage 3) was characterised by an increase in creatinine 3 times baseline or to ≥4.0 mg/dl). To simplify our statistical analysis because of the low occurrence rate, AKI stages 2 and 3 were combined into a single category (2–3), leaving us with three final groups (no AKI vs AKI stage 1 vs AKI stage 2–3).

Long-term renal injury was staged according to similar thresholds adopted from the KDIGO criteria. This was defined using the difference between the preoperative creatinine concentration and the long-term follow-up measurement as follows: no renal injury (creatinine increase of less than 0.3 mg/dl and less than 1.5 times the baseline); mild injury (creatinine increase of at least 0.3 mg/dl or 1.5 to 1.9 times the baseline level); moderate injury (creatinine 2.0 to 2.9 times the baseline); or severe injury (increase to greater than 3.0 times baseline or creatinine level of at least 4 mg/dl or dependency on renal replacement therapy).

### Statistical analysis

Distribution of continuous data was analysed using a Kolmogorov-Smirnov test. Normally distributed data are presented as means ± standard deviation and were compared between groups using a one-way analysis of variance. Non-normally distributed data are presented as medians (interquartiles ranges) and were compared using a Kruskall-Wallis test. Dichotomous variables are presented as crude numbers and percentages and were compared between groups using a Chi-square test. Modelling of the risk of long-term renal injury was performed using the same approach as Turan et al. [20] including early AKI and all covariates listed in Tables 1 and 2 in a logistic (binomial) model. Criteria which were independently associated with AKI in a univariable model at *P* < 0.05 were included in the multivariate model. For factors that were considered by the authors to be clinically equivalent measures of the same feature (for example weight and BMI, or Child-Pugh score and Child-Pugh class), only the factor with the strongest significance was included. The risk of developing long-term renal injury is presented as an odds ratio with 95% confidence intervals. Statistical analyses were done using Minitab 16 (Paris, France and Medcalc Software LTD, Ostend, Belgium) and R (www.r-project.org).

**Table 1:** Patient Characteristics by acute kidney injury status

Variables	No AKI (*N* = 706)	AKI stage 1 (*N* = 81)	AKI stage 2–3 (*N* = 28)	*P*-value
Age (years)	65 [55–72]	68 [63–74]	65 [57–75]	**0.038**
Male (%)	424 (60)	60 (74)	25 (89)	**0.001**
BMI (kg/m^2^)	25 [23–29]	26 [22–30]	27 [25–35]	0.1
ASA score (1–2 / 3–4)	430 / 276	50 / 31	16 / 12	0.9
Preop Hb (g/dL)	13.3 [12–14.5]	13.4 [11.8–14.3]	13.9 [12.5–14.7]	0.7
Preop creatinine (mg/dL)	0.8 [0.7–1.0]	1.0 [0.8–1.1]	1.0 [0.8 1.2]	**< 0.001**
***Comorbidities; N (%)***				
Ischemic heart disease	60 (8.5%)	3 (3.7%)	9 (32%)	**< 0.001**
Coronary arterial bypass graft	28 (3.9%)	3 (3.7%)	3 (11%)	0.2
Hypertension	319 (45%)	52 (64%)	20 (71%)	< **0.001**
Hyperlipidaemia	194 (27%)	28 (35%)	7 (25%)	0.4 0.2
Stroke	28 (3.9%)	2 (2.5%)	3 (11%)	**0.01**
Atrial fibrillation	44 (6.2%)	12 (15%)	1 (3.6%)	0.9
Diabetes mellitus type 2	151 (21%)	17 (21%)	7 (25%)	**0.008**
COPD	86 (12%)	10 (12%)	9 (32%)	0.7
Cirrhosis	53 (7.5%)	4 (4.9%)	2 (7.1%)	
***Medications; N (%)***				
Aspirin	236 (33%)	32 (40%)	8 (29%)	0.5
Clopidogrel	32 (4.5%)	1 (1.2%)	3 (11%)	0.1
ẞ blocker	166 (24%)	28 (35%)	13 (46%)	**0.003**
ACEI	135 (19%)	23 (28%)	11 (39%)	**0.007**
ARB	47 (6.7%)	8 (9.9%)	3 (11%)	0.4
Calcium channel blocker	113 (16%)	19 (23%)	7 (25%)	0.1
Diuretics	59 (8.4%)	8 (9.9%)	8 (29%)	**0.001**
Statin	211 (30%)	18 (22%)	12 (43%)	0.1
Oral hypoglycaemic drugs	111 (15.7%)	10 (12%)	4 (14%)	0.7
Insulin	56 (7.9%)	7 (8.6%)	2 (7.1%)	0.9
Oral anticoagulation	65 (9.2%)	9 (11%)	3 (11%)	0.8
***Type of Surgery (N)***				**0.02**
Pancreatectomy	155 (22%)	18 (22%)	3 (11%)	
Hepatobiliary	189 (27%)	14 (17%)	3 (11%)	
Oesophagectomy	75 (11%)	12 (15%)	3 (11%)	
Cystectomy	63 (8.9%)	15 (19%)	7 (25%)	
Cancer debulking	32 (4.5%)	2 (2.5%)	2 (7.1%)	
Major aortic vascular surgery	144 (20%)	15 (19%)	9 (32%)	
Other surgical procedure *	48 (6.7%)	5 (6.2%)	1 (3.6%)	

Values are presented as medians [interquartiles ranges] or numbers (percentages %)

*Abbreviation*: *COPD* chronic obstructive pulmonary disease, *AKI* acute kidney injury, *BMI* body mass index, *preop* preoperative, *ACEI* Angiotensin-converting-enzyme inhibitor, *ARB* Angiotensin II receptor blocker, *Hb* haemoglobin, *ASA* American Society of Anesthesiologists

* included: gastrectomy, open colectomy nephrectomy, surrenalectomy, prostatectomy)

**Table 2:** Intraoperative variables by acute kidney injury status

Variables	No AKI (*N* = 706)	AKI stage 1 (*N* = 81)	AKI stage 2–3 (*N* = 28)	*P*-value
Anaesthesia duration (min)	346 [260–446]	421 [339–502]	451 [354–576]	**< 0.001**
Surgery duration (min)	262 [184–352]	337 [263–397]	366 [282–445]	**< 0.001**
Total crystalloid (ml)	2000 [1300–3000]	3000 [2000–4000]	4000 [2000–5900]	**< 0.001**
Total colloid ^**£**^ (ml)	500 [500–1000]	1000 [500–1500]	1000 [500–2000]	**0.008**
Total blood product (ml)	500 [300–800]	550 [396–1700]	525 [270–1900]	0.2
Total IN (ml)	2500 [1800–3500]	3500 [2500–5400]	4500 [2300–7300]	**< 0.001**
Estimated blood loss (ml)	500 [200–1000]	700 [300–1700]	1000 [500–1600]	**< 0.001**
Diuresis (ml)	300 [150–500]	300 [200–500]	300 [200–500]	0.9
Gastric suction (ml)	100 [50–100]	50 [50–100]	50 [50–160]	0.7
TOTAL OUT (ml)	900 [500–1600]	1100 [700–2100]	1400 [900–2200]	**0.002**
Net fluid balance (ml)	1500 [800–2300]	2300 [1400–3300]	3100 [1100–5200]	**< 0.001**
Use of vasopressors, $ N (%)	554 (78%)	67 (83%)	23 (82%)	0.6

Values are presented as medians [interquartiles ranges] or numbers (percentages %)

**$**: use of any vasopressor (ephedrine, phenylephrine, noradrenaline)

^**£**^ total colloid included 3% gelatin and 6% tetrastarch

## Results

**Fig. 1:**
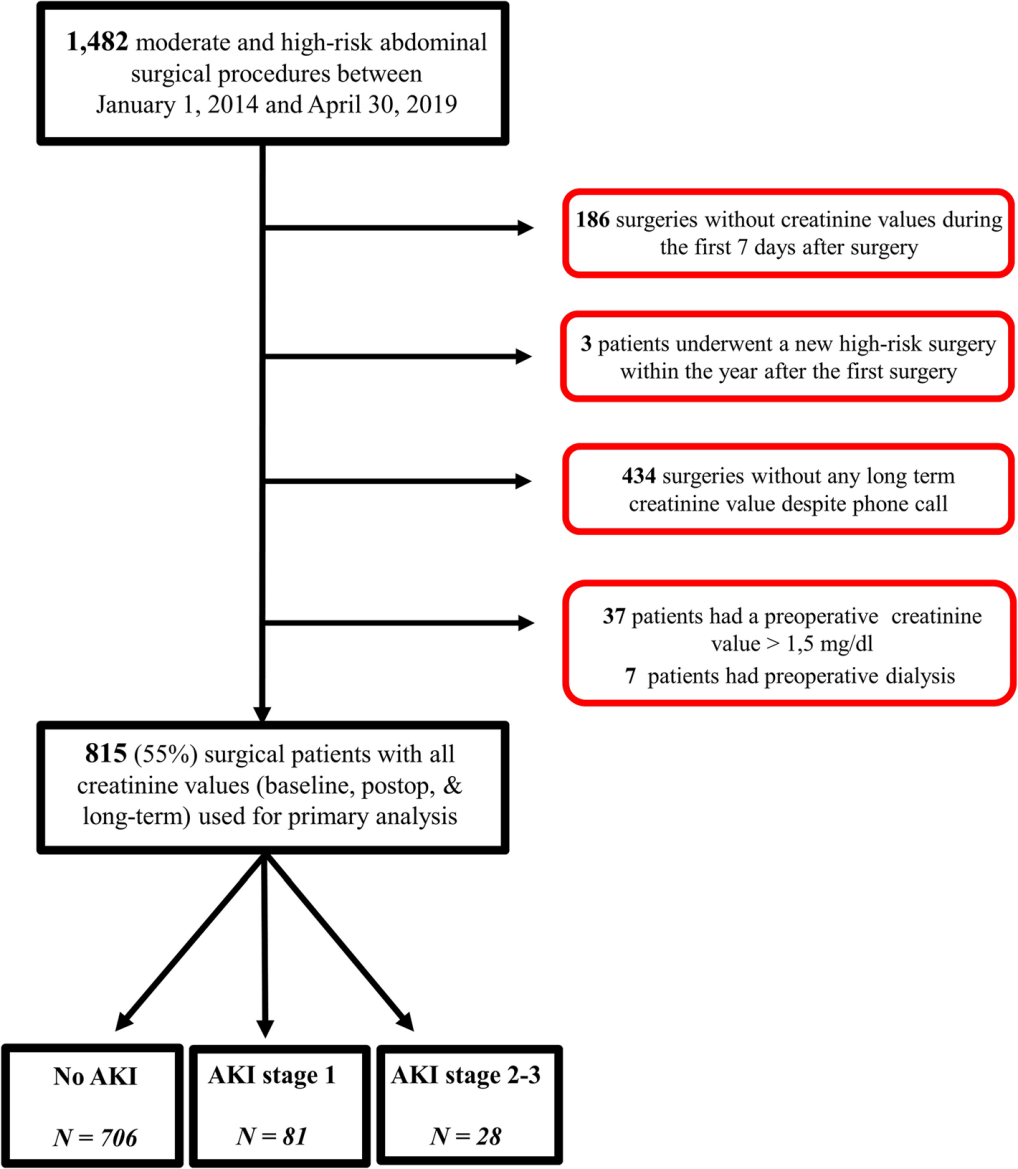
Enrollment flow chart. AKI: acute kidney injury

Among the 1482 patients who underwent intermediate-to high-risk abdominal surgery between January 1st 2014 and April 30th 2019, 815 patients met the inclusion criteria and were thus included in our study. The main reason for exclusion was lack of postoperative or long-term creatinine values **(**Fig. 1**).**

**Fig. 2:**
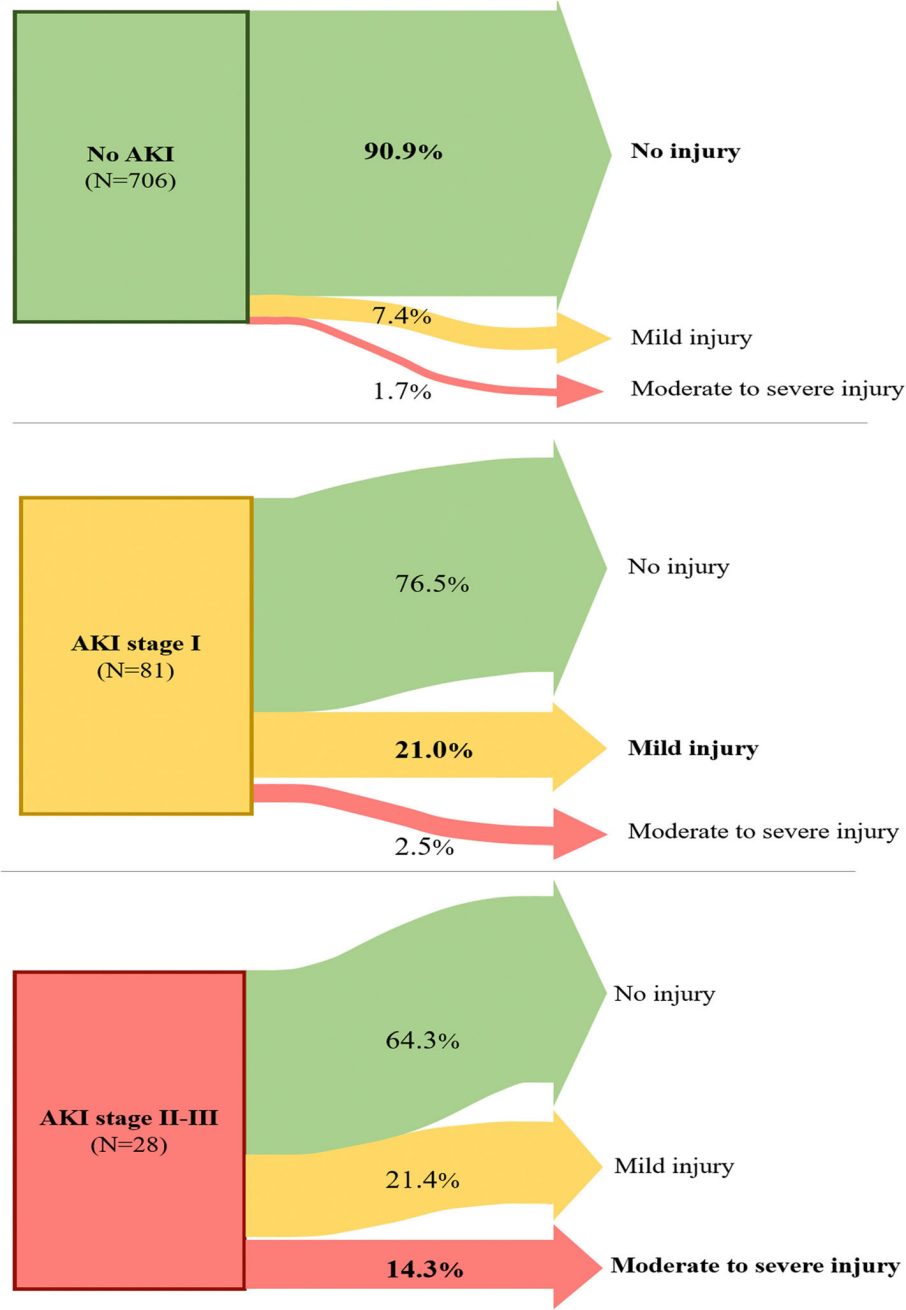
Renal outcomes 1 y after surgery according to postoperative acute kidney injury (AKI) stage

Among the 81 patients who developed mild postoperative AKI, 19 patients (24%) had persistent mild or moderate-to-severe renal injury 1 y after surgery, compared to 64 (9.1%) of those who had no postoperative AKI (*P* < 0.001) **(**Fig. 2**).** Among the 28 patients (3.4%) who developed moderate to severe AKI postoperatively, 10 (36%) had some degree of long-term renal dysfunction. Patients who developed mild AKI after surgery therefore had a threefold higher chance of developing long-term renal injury compared to patients without postoperative AKI (odds ratio [95% CI] of 3.1 [1.7–5.5]; *P* = 0.0001). In patients with postoperative AKI KDIGO stage 2–3, the odds ratio for development of long-term renal dysfunction was 5.6 (95% CI 2.5–12.6; *P* < 0.0001).

Occurrence of postoperative AKI was associated with older age, higher baseline creatinine level and presence of comorbid conditions, notably a history of chronic hypertension, myocardial infarction, atrial fibrillation, or chronic obstructive pulmonary disease (Table 1). Patients who developed postoperative AKI had longer surgery times, received more fluid and had a higher estimated blood loss during surgery compared to patients without postoperative AKI (Table 2). Patients with postoperative AKI also had more postoperative complications and a longer hospital length of stay (Table 3). Importantly, long-term creatinine values were measured around one year in median among all groups (Table 3; *P* = 0.190).

**Table 3:** Postoperative Variables by acute kidney injury status

Variables	No AKI (*N* = 706)	AKI stage 1 (*N* = 81)	AKI stage 2–3 (*N* = 28)	*P*-value
Length of stay in hospital (days)	9 [6–14]	15 [9–28]	17 [9–28]	**< 0.001**
Creatinine max during the first POD#7 (mg/dl)	0.8 [0.7–1.0]	1.4 [1.2–1.7]	2.8 [1.8–3.8]	**< 0.001**
Creatinine at hospital discharge	0.7 [0.6–0.9]	1.0 [0.8–1.2]	1.1 [0.8–1.5]	**< 0.001**
Creatinine at long-term follow up	0.9 [0.7–1.0]	1.0 [0.9–1.2]	1.1 [0.9–1.4]	**< 0.001**
Measurement of long-term follow up creatinine (days after surgery)	360 [303–393]	354 [298–376]	353 [294–386]	0.2
**Minor complications;** ***N*** **(%)**	146 (21%)	36 (44%)	14 (50%)	**< 0.001**
Superficial wound infection	19 (2.7%)	3 (3.7%)	0 (0%)	
Urinary infection	33 (4.7%)	10 (12%)	5 (18%)	
Paralytic ileus	20 (2.8%)	4 (4.9%)	5 (18%)	
Pneumonia	13 (1.8%)	1 (1.2%)	2 (7.1%)	
Postoperative confusion	20 (2.8%)	4 (4.9%)	3 (11%)	
Other infection	72 (10%)	20 (25%)	7 (25%)	
**Major complications;** ***N*** **(%)**	101 (14)	27 (33)	11 (39)	**< 0.001**
Anastomotic leakage	19 (2.7%)	5 (6.2%)	2 (7.1%)	
Peritonitis	4 (0.57%)	1 (1.2%)	1 (3.6%)	
Sepsis	22 (3.1%)	13 (16%)	5 (18%)	
Necrosis stoma	8 (1.1%)	1 (1.2%)	0 (0%)	
Wound dehiscence	9 (1.3%)	2 (2.4%)	1 (3.6%)	
Bleeding requiring a redo surgery	24 (3.4%)	7 (8.6%)	1 (3.5%)	
Pulmonary embolism	5 (0.71%)	1 (1.2%)	0 (0%)	
Pulmonary oedema	7 (0.99%)	1 (1.2%)	1 (3.6%)	
Acute coronary syndrome	0 (0%)	1 (1.2%)	0 (0%)	
Atrial fibrillation / arrhythmia	15 (2.1%)	4 (4.9%)	1 (3.6%)	
Stroke	0 (0%)	0 (0%)	0 (0%)	
Renal replacement therapy	0 (0%)	0 (0%)	0 (0%)	
Reoperation	35 (4.9%)	10 (12%)	3 (11%)	
30-day mortality	0 (0%)	0 (0%)	0 (0%)	

Values are medians [interquartiles rangees] or numbers (percentages %)

In multivariable analysis, development of postoperative AKI was independently associated with long-term renal injury (adjusted odds ratio [95%CI] of 4.5 [1.8–11.4]; *P* = 0.002) (Table 4).

**Table 4:** Significant independent variables predicting long-term kidney injury in multivariable model

Variables	Adjusted odds ratio	[95%CI]	*P*-value
Postop AKI	4.5	[1.8–11]	0.002
ASA score	10.9	[1.2–100]	0.036
ARB	0.07	[0.0–0.7]	0.027
LLAO	4.9	[1.1–22]	0.037
Hepatectomy	0.12	[0.02–0.67]	0.016

*ASA* American Society of Anesthesiology physical status

*ARB* angiotensin II receptor blockers

*LLAO* lower limb arteriopathy obliterans

## Discussion

In a cohort of 815 patients who underwent intermediate-to high-risk abdominal surgery, more than one fifth of patients (21%) who developed mild postoperative AKI had mild renal injury long term, and 2.5% developed moderate to severe long-term renal injury. This observation demonstrates that even a slight increase in postoperative creatinine can be important and should not be neglected. Stated a different way, development of mild postoperative AKI more than tripled the odds of having renal injury 1 y after surgery compared to patients without postoperative AKI.

Our results are in agreement with the only available study which examines the association of mild AKI with long-term renal injury [20]. This study, recently published by Turan et al., utilized a large database from the Cleveland Clinic that included more than 15,000 patients who underwent a variety of non-cardiac surgical procedures ranging from low to high-risk. Interestingly, postoperative AKI was a complication in only 3% of their study population compared to 13.4% in our study. This is not surprising as we included only patients who had had high-risk abdominal surgery that carries a greater risk of postoperative renal dysfunction than low-risk abdominal surgery. Moreover, major surgery is a well-known contributing factor for postoperative AKI [22]. This increased risk is likely due to larger fluid shifts, blood losses and a relatively high incidence of perioperative hypotension in these patients, all of which can compromise renal blood flow [23–26]. Moreover, these types of surgical procedure are more often performed in elderly patients who are at a greater risk of having comorbidities that predispose to development of AKI.

As in the study by Turan et al., we used the KDIGO classification system to enable us to compare short and long-term renal function. Our study design allowed inclusion of all patients who had a creatinine measurement between six months and two years after surgery. However, the timing of the long-term measurement did not differ between the three groups and was very close to one year. Another recent study published by Mizota et al. demonstrated that even transient AKI after major abdominal surgery increased the risk of chronic kidney disease and one-year mortality [19]. Thus, even if a patient recovers from AKI within the first week after surgery, they should be considered at a greater risk of worse long-term outcome.

This study has some limitations that should be taken into consideration. First, our study was observational, retrospective, and single-centre and included a relatively small sample size, largely because of the high proportion of patients without long-term follow up creatinine concentrations. Therefore, a causal relation cannot be proven. Second, we excluded patients using a maximum creatinine cut-off value of 1.5 mg/dl and not using estimated glomerular filtration rate. Third, we were unable to determine if the long-term creatinine value that was obtained was collected in a stable patient or in someone experiencing a new onset acute illness. Fourth, we only included patients who underwent moderate to high-risk open abdominal surgery [27] so that the data cannot be extrapolated to other types of surgery (neurosurgical, cardiac, etc). Moreover, we did not exclude the 9 patients (~ 1%) who underwent an open nephrectomy as this small number likely has a negligible impact on the study results. Fifth, as urine output was not used for the AKI classification (“modified” KDIGO classification), this may have led to an “underestimation” of the incidence of postoperative AKI in our study cohort. Lastly, our logistic regression only took into account perioperative variables so that we did have information on specific events during the long-term follow-up (oncological evolution or cardiovascular problems).

## Conclusions

Although mild increases in postoperative plasma creatinine concentration are frequently considered to have little long-term clinical significance, we found that patients undergoing intermediate-to high-risk abdominal surgery who developed a mild increase in plasma creatinine concentration had a much higher incidence of long-term renal dysfunction. Clinicians should not neglect “minor” disturbances in renal function after surgery as they may persist or even worsen during long-term follow up. The presence of even mild postoperative AKI may indicate a need for tighter follow up to monitor long-term renal function.

## Data Availability

By request to the corresponding author.

## Abbreviations

KDIGO: Kidney Disease: Improving Global Outcomes
AKI: Acute kidney injury
