# Correction: Mild increases in plasma creatinine after intermediate to high-risk abdominal surgery are associated with long-term renal injury

**DOI:** 10.1186/s12871-024-02556-z

**Published:** 2024-05-23

**Authors:** Alexandre Joosten, Brigitte Ickx, Zakaria Mokhtari, Luc Van Obbergh, Valerio Lucidi, Vincent Collange, Salima Naili, Philippe Ichai, Didier Samuel, Antonio Sa Cunha, Brenton Alexander, Matthieu Legrand, Fabio Silvio Taccone, Anatole Harrois, Jacques Duranteau, Jean-Louis Vincent, Joseph Rinehart, Philippe Van der Linden

**Affiliations:** 1 Department of Anesthesiology, CUB ErasmeUniversité Libre de Bruxelles, 808 Route de Lennik, Bruxelles, 1070 Belgium; 2 Department of Anesthesiology and Intensive Care, Hôpitaux Universitaires Paris-SudUniversité Paris-Sud, Paul Brousse Hospital, Assistance Publique Hôpitaux de Paris (APHP), Université Paris-Saclay, 12 Avenue Paul Vaillant Couturier, Villejuif, 94800 France; 3 Department of Hepato-Biliary Surgery, CUB ErasmeUniversité Libre de Bruxelles, 808 Route de Lennik, Bruxelles, 1070 Belgium; 4 Department of Anesthesiology, Médipole, Lyon Villeurbanne, France; 5 Department of Liver Intensive Care Unit, AP-HP, Assistance Publique Hôpitaux de Paris, Paul-Brousse Hospital, Centre Hépato-Biliaire, 12 Avenue Paul Vaillant Couturier, Villejuif, 94800 France; 6 Department of Hepato-Biliary and Pancreatic Surgery, Assistance Publique Hôpitaux de Paris, Paul-Brousse Hospital, Centre Hépato-Biliaire, 12 Avenue Paul Vaillant Couturier, Villejuif, 94800 France; 7 Department of Anesthesiology, University of California San Diego, 9500 Gilman Dr, La Jolla, CA 92093 USA; 8 Department of Anesthesia and Perioperative Care, University of California, San Francisco, 500 Parnassus Avenue, San Francisco, USA; 9 UMR INSERM 942, Institut National de La Santé Et de La Recherche Médicale (INSERM), INI-CRCT Network, Paris, France; 10 Department of Intensive Care, CUB Erasme, Université Libre de Bruxelles, 808 Route de Lennik, Bruxelles, 1070 Belgium; 11 Department of Anesthesiology and Intensive Care, Hôpitaux Universitaires Paris-SudUniversité Paris-SudBicetre Hospital, Assistance Publique Hôpitaux de Paris (APHP), Université Paris-Saclay, 78 Rue du General Leclerc, Le Kremlin Bicetre, 94270 France; 12 Department of Anesthesiology and Perioperative Care, University of California Irvine, 101, the City Drive South, Orange, CA USA; 13 Department of Anesthesiology, Brugmann Hospital, Université Libre de Bruxelles, 4, Place A. Van Gehuchten, Bruxelles, 1020 Belgium

**Correction** **:** **BMC Anesthesiol 21, 135 (2021)**

**https://doi.org/10.1186/s12871-021-01353-2**

Following publication of the original article [1], the authors identified an error in the author name of Zakaria Mokhtari.

The incorrect author name is: Zakaria Mokthari

The correct author name is: Zakaria Mokhtari

The author group has been updated above and the original article [1] has been corrected.
